# **Comment on:** “Pregnancy Outcomes After Bariatric Surgery: Importance of Maternal Ferritin on Birth Weight”

**DOI:** 10.1007/s11695-024-07286-2

**Published:** 2024-06-06

**Authors:** Maite Aguas-Ayesa, Javier Gómez-Ambrosi, Gema Frühbeck

**Affiliations:** 1 Department of Endocrinology and Nutrition, Clínica Universidad de Navarra, Avda. Pío XII 36, 31008 Pamplona, Spain; 2 CIBER Fisiopatología de La Obesidad y Nutrición (CIBEROBN), ISCIII, 31008 Pamplona, Spain; 3 Obesity and Adipobiology Group, IdiSNA, 31008 Pamplona, Spain; 4 Metabolic Research Laboratory, Clínica Universidad de Navarra, 31008 Pamplona, Spain

Of all the available therapies, in adequately selected patients, bariatric surgery (BS) is the most effective treatment for severe obesity and its complications [1]. Despite the numerous benefits of BS, even in reproductive performance, women who have undergone BS have higher rates of small-for-gestational age (SGA) fetuses and preterm births than non-intervened women with the same body mass index (BMI) [2]. The mechanisms involved are not known, but nutritional deficiencies, common after BS [3], have been identified as one of the factors that could contribute to these pregnancy complications [4]. In this issue of *Obesity Surgery*, Lecot-Conann and colleagues [5] present an interesting retrospective, single-center observational cohort study based on 120 French pregnant women (mean age 32.6 years [SD 5.3] with a history of prior BS (61% gastric bypass, 18% adjustable gastric banding, 15% sleeve gastrectomy, and 6% one anastomosis gastric bypass) that had at least one nutritional assessment during pregnancy that included hemogram, ferritin, calcium, 25OH vitamin D, parathyroid hormone, fasting glucose, albumin, prealbumin, vitamin A, vitamin B12, folic acid, and zinc to analyze maternal and neonatal outcomes as well as their possible association with the nutritional variables evaluated.

The authors showed that ten (8%) women gave birth prematurely while 22 (19%) newborns were SGA. The study adds to the already existing evidence that SGA and preterm birth are more prevalent after BS than in non-intervened women. This finding has relevant implications given the potential link with the development of obesity later in adulthood [6]. However, the prevalence of SGA and preterm birth in previously published studies is highly variable [2]. Interestingly, in the French study, mothers with SGA babies exhibited ferritin levels significantly higher than those giving birth to babies with adequate size (35.5 [22.3–69.5] vs. 15 [10–32] ng/mL) in the third trimester of pregnancy [5], which lead the authors to suggest that iron supplementation should be used with caution in these women. The results are compatible with those found by Coupaye and colleagues [7], who observed increased ferritin levels in neonates with SGA from women who underwent BS.

In the present study, Lecot-Conann and colleagues [5] explored a wide number of factors that could be related to the high rates of SGA and preterm births finding no association with time from BS or maternal characteristics including pregestational BMI, smoking habits, nulliparity, and gestational weight gain. Regarding the interval between surgery and conception, most studies have not found a relationship between this factor and SGA or preterm birth [2]. In the same line, published results regarding the effect of preoperative BMI are also inconclusive, although a higher preoperative BMI seems to have a protective role for SGA and preterm birth. Lecot-Connan et al. [5] observe that there is no evidence supporting the association of SGA and prematurity with tobacco use in agreement with previous studies [2].

Another important factor that the authors evaluated was the possible relationship with the type of surgery showing that women who underwent adjustable gastric banding and sleeve gastrectomy had a higher tendency to have SGA neonates without reaching statistical significance in comparison with the other surgical procedures [5]. Current knowledge does not allow to establish a clear relationship between the type of surgery and the rate of SGA and prematurity, although it seems that surgeries with a malabsorptive component could lead to higher rates of SGA [2].

The study by Lecot-Conann and colleagues [5] also highlights the analysis of the influence of nutritional education finding a tendency towards a lower rate of SGA neonates in those women who had received pre-pregnancy nutritional counseling, although without statistical significance. This factor has been scarcely studied, but periodic nutritional counseling during pregnancy has been related to lower levels of SGA and preterm birth [2, 8].

The study by Lecot-Connan et al. [5] has some limitations though, like the relatively small sample size which probably explained the limited statistical power. Furthermore, the results obtained were not compared with a sample of BMI-matched pregnant women that had not undergone BS. It would have been particularly interesting to compare ferritin levels between the mentioned groups since people with obesity usually exhibit higher levels than people with normal weight. On the other hand, it would have been interesting to know previous ferritin levels and the potential presence of fatty liver disease, since this is a factor that influences circulating ferritin concentrations [9].

In the context of an area that is still unclear, the data provided by Lecot-Conann and colleagues [5] are essential to increase knowledge about factors that could be involved in higher rates of SGA and preterm births after BS. Chronic inflammation and oxidative stress may operate as a link between elevated circulating ferritin concentrations, which may contribute to tissue or cellular damage [10]. However, the fact that the studies published in this area are small and have very heterogeneous designs makes it difficult to obtain conclusive information. Thus, it is mandatory to obtain more rigorous data through the design of larger multicenter studies. Robust information will allow to clarify the mechanisms involved in SGA and preterm births in pregnant women after BS and the development of strategies, within the context of precision medicine including evaluation protocols and supplementation strategies, to reduce these rates and improve outcomes.
